# Human Monkeypox: Current State of Knowledge and Implications for the Future

**DOI:** 10.3390/tropicalmed1010008

**Published:** 2016-12-20

**Authors:** Katy Brown, Peter A. Leggat

**Affiliations:** 1 College of Public Health, Medical and Veterinary Sciences, James Cook University, Townsville QLD 4811, Australia; peter.leggat@jcu.edu.au; 2 Médecins Sans Frontières, Geneva 1202, Switzerland

**Keywords:** human monkeypox, monkeypox, MPXV, MPX

## Abstract

The zoonosis human monkeypox (MPX) was discovered in 1970, twelve years after the discovery of monkeypox virus (MPXV) in a Danish laboratory in 1958. Historically confined to West Africa (WA) and the Congo basin (CB), new epidemics in Sudan and the United States of America (USA) have fuelled new research highlighting environmental factors contributing to the expanded geographical spread of monkeypox virus (MPXV). A systematic literature review was conducted in MEDLINE^®^ (Ovid), MEDLINE^®^ (PubMed) and Google Scholar databases using the search terms: monkeypox, MPXV and “human monkeypox”. The literature revealed MPX has classic prodromal symptoms followed by a total body rash. The sole distinguishing clinical characteristic from other pox-like illnesses is the profound lymphadenopathy. Laboratory diagnosis of MPX is essential, a suitable test for endemic areas is under development but not yet available. For the time being anti-poxvirus antibodies in an unvaccinated individual with a history of severe illness and rash can suggest MPX infection. The reservoir host remains elusive yet the rope squirrel and Gambian pouched rat appear to be the most likely candidates. Transmission includes fomite, droplet, direct contact with infected humans or animals and consumption of infected meat. Though smallpox vaccination is protective against MPXV, new non-immune generations contribute to increasing incidence. Environmental factors are increasing the frequency of contact with potential hosts, thus increasing the risk of animal-to-human transmission. Increased risk of transmission through globalisation, conflict and environmental influences makes MPX a more realistic threat to previously unaffected countries. Health worker training and further development and accessibility of suitable diagnostic tests, vaccinations and anti-viral treatment is becoming increasingly necessary.

## 1. Introduction

Following the eradication of smallpox in 1980, human monkeypox (MPX) was described in 1987 as the most important orthopoxvirus (OPXV) occurring in humans at the time [1]. There are currently 10 species known in the genus OPXV, including variola (smallpox) [1]. With a 30% case fatality rate (CFR) and high virulence, smallpox is described as one of the most feared diseases known to humanity [2]. Monkeypox virus (MPXV) is highly pathogenic [3,4], causing similar clinical manifestations to smallpox. Smallpox vaccination is known to provide cross-immunity with up to 85% protection against infection [5,6] or reduction in severity of disease [7,8,9,10]. 

MPXV was first discovered during an outbreak amongst monkeys at a Danish laboratory in 1958 [11]. However, it was not recognised as a human disease until 1970, when a nine-month-old child became infected in Democratic Republic of Congo (DRC), formerly known as Zaïre [12]. MPX is typically found in the tropical rain forests of the Congo basin (CB) and West Africa (WA) [7], and DRC continues to report the majority of cases each year [13,14], mainly in children under 10 years [15]. The latest epidemic was in Central African Republic (CAR) in October 2016, resulting in 26 cases, of which three were laboratory confirmed [16,17].

**Figure 1:**
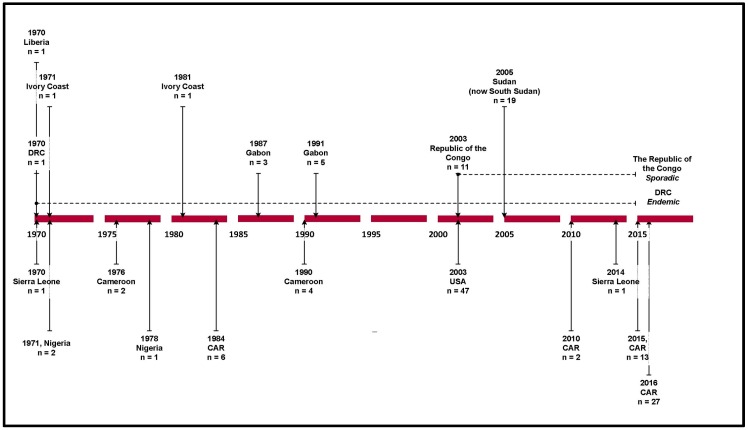
Timeline of reported human monkeypox outbreaks. Source: based on data from Centers for Disease Control and Prevention [6], Formenty et al. (2010) [21], Learned et al. (2005) [22], International Federation of Red Cross and Red Crescent Societies (2016) [23], Damon et al. 2006 [24].

In 2003 the first MPX outbreak to occur outside of Africa was reported in the United States of America (USA), after 800 small African mammals were shipped from Ghana into Texas [11,15,18,19]. Amongst the shipment were three rope squirrels, two giant pouched rats, and nine dormice infected with MPXV [20]. The infected rodents were sold to wholesale distributers, where they were kept in close proximity with American native prairie dogs (PD). Thereupon the PD became infected with MPXV prior to being sold to a second animal distributor [11,15]. Subsequently MPX spread to five states with a total of 47 cases [6,15] triggering an importation ban on all African rodents into the USA [5,6]. The Sudanese outbreak of 2005 constitutes the second epidemic of MPX recorded outside of the CB and WA regions [21] (See Figure 1 for outbreak timeline).

MPXV is a brick-shaped enveloped virus measuring 200–250 nm, which replicates in the cytoplasm, not the nucleus [14]. Two clades of MPXV have been identified through genomic sequencing: WA and CB clades [15]. WA and CB MPXV differ in virulence [11,18] and are genetically distinct [25]. It is suggested that WA MPXV is attenuated and less transmissible than CB [14,26,27]. However, differences in disease severity may also be affected by transmission route, host susceptibility, and the quantity of virus inoculated [18]. The dose required to induce clinical disease varies considerably. MPXV is a typical zoonosis in that the majority of documented infections are from an animal source [5,28,29]. During the WHO active surveillance program in DRC between 1981 and 1986, 72% of the MPX cases were found to be zoonotic transmission [28]. Antibodies to MPXV have been detected in multiple animal species, suggesting the natural lifecycle is a complex interaction of reservoir and incidental hosts [9,11]. Though MPXV is not as virulent as variola virus [14], human-to-human transmission is possible [22]; humans are considered to be incidental hosts [14].

The USA epidemic was the first opportunity to study MPX outside of Africa [8] and the largest case series of WA clade MPX [30]. Almost five decades after the discovery of MPX, multiple aspects of MPXV require clarification, including: the ecology of the virus in wildlife, a complete picture of mode of transmission from wildlife to humans, and the extent of person-to-person transmission [7]. Despite an abundance of new information, these fundamental elements remain unanswered.

This paper reviews the current state of knowledge of MPX, with emphasis on clinical features, transmission, diagnosis and prevention. Discoveries in light of the USA epidemic and critical issues impacting the future of MPX are identified. 

## 2. Materials and Methods 

**Figure 2:**
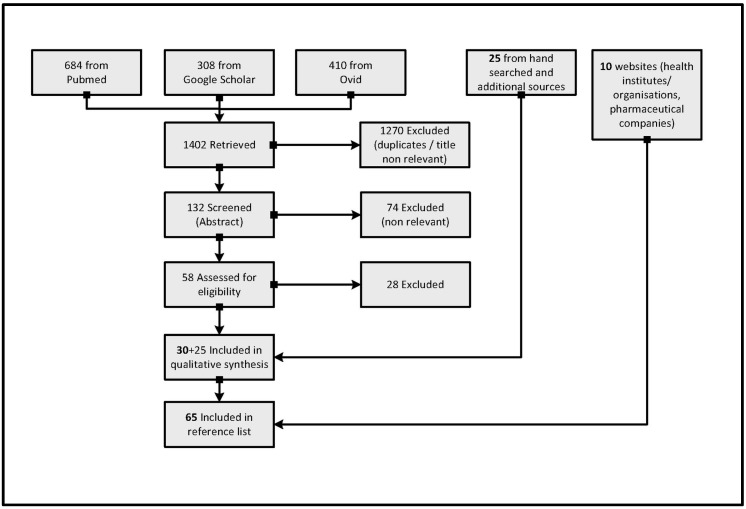
Flow diagram of systematic review. Source: based on PRISMA statement [31].

A systematic literature search was conducted in MEDLINE^®^ (Ovid), MEDLINE^®^ (PubMed) and Google Scholar databases. The following search terms and Boolean operators were used: monkeypox OR MPXV OR “human monkeypox”. No date range was specified in the initial Pubmed search in order to provide historical perspective and capture initial research. The search terms were then modified by applying the date limits 2000–2016 when searching in the remaining databases. The motivation for doing so was to focus on contemporary studies conducted since the 2003 USA epidemic. The search was not limited to human studies as MPXV is a zoonosis. Moreover, no language limits were set as many countries of interest are French speaking, although all retrieved articles were in English. After removal of duplicates and non-relevant papers according to title, 132 papers remained for screening of abstract. As with the title screen, articles were excluded if the abstract content was out of the scope of this paper in terms of subject specialisation and narrow subject focus; 74 were removed at this stage. Furthermore, editorial and correspondence pieces were excluded. Therefore, of the 58 full-text articles assessed for eligibility, six were rejected for virology and immunology specialisation; 16 due to study quality, and six due to inaccessibility (Figure 2). Ultimately 30 articles remained, which were complemented with a further 25 papers from hand searches of reference lists and additional sources. Finally 10 seminal and supporting websites were cited.

## 3. Results

### 3.1. Clinical Features

**Figure 3:**
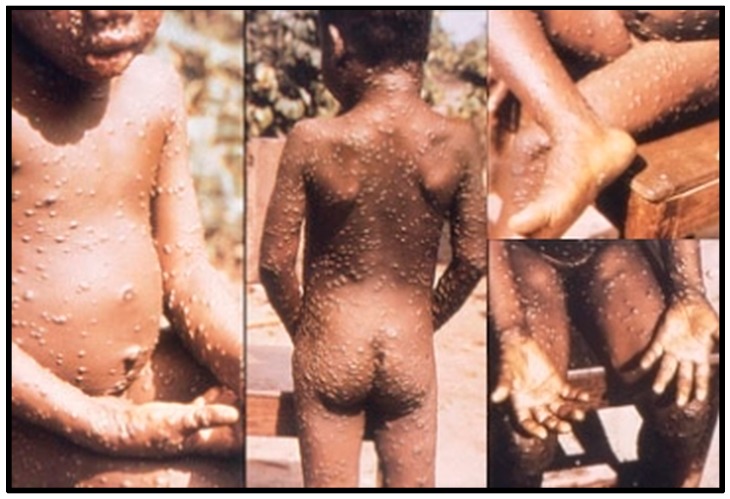
Monkeypox rash. Source: Centers for Disease Control and Prevention [6].

The most prevalent clinical features of MPX are shown in Table 1. Prodromal symptoms lasting 2–4 days include fever, fatigue and lymphadenopathy, accompanied by some headache and backache [7,14,15,19]. Fever reduces 3 days after the onset of a smallpox-like rash, which begins on the face and quickly spreads centrifugally over the body [7,13], including oral mucosa, genitalia, and palms and soles, as shown in Figure 3 [13,14,21]. The rash lasts approximately 2–4 weeks, starting as sequentially forming macules, which transform into papules, vesicles, pustules and finally crusts [14,32,33,34].

Ranging from 0.5 to 1 cm in diameter and up to several thousand in number [7], the pustules become centrally depressed, scab (crust) and then desquamate [5,14,33], which takes around 12 days [19]. It has been generally accepted the onset of rash marked the onset of infectious period [6,10,36,37]. However the Centers for Disease Control and Prevention (CDC) state that a person may sometimes be contagious during the prodromal period [6]. The incubation period is approximately 12 days [14,15], but can be up to 21 days [6].

In 1987 pronounced lymphadenopathy was identified as the only clinical sign differentiating MPX from smallpox and chickenpox (varicella) [5,32,35]. Although lymphadenopathy remains a key distinguishing feature, the appearance and evolution of lesions in chickenpox is markedly different. Chickenpox lesions tend to be more superficial, smaller and unlike the centrifugal distribution of MPX they are centrally located and evolve in ‘crops’ over 3–5 days, compared to the average 12 days for MPX [34]. Thus a slower maturation of skin lesions is an important differentiation when analyzing skin lesions [7]. It should be noted that not all MPX cases present with multiple lesions. In the USA epidemic, a 28-year-old female who had direct contact with an infected PD and went on to develop prodromal symptoms followed by lymphadenopathy (and later tested positive for MPXV in serologic testing) presented with only one lesion [19,38]. Furthermore, no ‘crust’ stage was described by the patient [38]. This case highlights the weakness of clinical recognition alone (a frequent reality for endemic countries) when diagnosing OPXV or differentials. In endemic countries, mucosal lesions or unusual eruptive skin rashes associated with pronounced lymphadenopathy, gastrointestinal symptoms and hematologic abnormalities, should include MPX in the differential diagnosis [35].

Complications of MPX include encephalitis [35,39] and severe dehydration secondary to vomiting and diarrhoea (or difficulty in drinking due to mouth lesions) [13,39]. Furthermore, tonsillitis, pharyngitis [7,19], oedema of the eyelids and conjunctivitis are common complications [7]. Respiratory symptoms are not referenced frequently (see Table 1), and are largely identified as a complication, such as bronchopneumonia [13]. The lasting effect of those who survive MPX is pitted scarring [6,13]. Moreover, corneal scarring can cause extensive and permanent damage to the eyes [22]. Patients in the USA had similar clinical features to African MPX cases but were milder in severity [5].

Biochemistry showed leucocytosis, raised transaminase levels, low blood urea nitrogen level and hypoalbuminemia [35]. Disease burden is high in Africa and multiple co-morbidities can depress immune response and increase vulnerability [9,14]. Approximately 20% of paediatric patients in the USA experienced serious complications that may have proved fatal if intensive treatment had been unavailable [35]. The CFR varies between epidemics; however the CDC places it at approximately 10% in Africa [6]. The most recent outbreak in CAR has documented two deaths of the 26 cases between August and October 2016 [17]—which would correspond to a CFR of 7.7%. Table 2 shows recorded CFRs from previous outbreaks.

### 3.2. Reservoir Host

Though the natural host remains unknown, the Gambian pouched rat and rope squirrel seem to be the most likely candidates [1,9,11,13,45]. Both species were amongst the infected rodents imported into the USA in 2003 [9,20,45]. Furthermore, the rope squirrel was identified as a reservoir host as early as 1985 [46]. This was reaffirmed by Thomassen et al. (2013), when they mapped the geographical distribution of MPX and potential reservoir hosts in DRC [9]. Recently, MPXV has been isolated in the sooty mangabey monkey (found dead in the Ivory Coast, 2012) [4]. This discovery is relatively new and as a result is not identified frequently within the literature but may prove vital in identification of the reservoir host (See Table 3).

### 3.3. Diagnosis

Rapid diagnosis is crucial to limiting outbreaks, but cannot be made on clinical observations alone [5,28]. MPXV can cause disease clinically indistinguishable from other pox-like illnesses, thus laboratory confirmation is essential [3,5]. The WHO identifies smallpox, chickenpox, measles, bacterial skin infections, scabies, medication allergies and syphilis amongst the differential diagnoses [47].

During the USA epidemic, laboratory evaluation of suspected MPX cases included PCR assays, electron microscopy, immunohistochemistry, culture of material from rash specimens, and serological testing for OPXV specific antibodies (Table 4) [30]. Unfortunately many countries burdened with MPX suffer from limited material resources for sample collection and storage, therefore point-of-care tests that can be used in very basic environments with limited training were needed. Since 2003 a rapid, point-of-care diagnostic (Tetracore Orthopox BioThreat Alert^®^) has been developed, particularly for field use [13,48]. In 2012 the first pilot of BioThreat Alert^®^ was conducted, concluding it was applicable as a point-of-care diagnostic for suspected MPX cases as well as a valuable screening tool to prioritize samples that required further testing [48]. The BioThreat Alert^®^ is the first lateral-flow based detection assay for OPXV [48], and although commercially available, there is currently no description of its use in MPX-endemic countries. 

Serological testing provides evidence of virus exposure, but this testing has limitations in diagnosis as it will detect immune responses to other OPXV exposures or vaccinations [5,30]. Studies have shown antiviral antibody and T-cell responses rise around the time of disease onset thus new highly-sensitive immunological techniques could improve diagnosis of MPX during an epidemic [8]. Meanwhile, anti-poxvirus antibodies in an unvaccinated individual with a history of severe illness and rash can suggest a diagnosis of MPX [28].

### 3.4. Transmission 

Table 5 displays the identified modes of transmission. The PD demonstrated high susceptibility to MPXV, becoming amplifying hosts infecting up to 47 people (none of the other imported animals infected humans) [11]. Discovery of MPXV in the lungs of PD was the first suggestion that transmission may have occurred via infective droplets [18]. Some USA patients were infected through existing wounds, others through bites or scratches from infected PD [15]. The invasive exposures (compared to fomite or droplet exposure) resulted in more severe systemic illness suggesting route of infection affects symptom severity [29]. Interestingly, eating infected bush meat or monkeys appears to be documented the least in terms of zoonotic transmission.

It is now confirmed MPXV is spread via exhaled large droplets [14], although these are unable to travel more than a few feet, thus prolonged close contact is required for human-to-human transmission [6,8]. Early studies believed human-to-human transmission of MPX was unsustainable and therefore not considered a serious public health threat [5,7]. However, an outbreak of CB MPXV in Republic of Congo in 2003 found six sequential passages of transmission [22]. Furthermore, a 2013 DRC study recently identified more than seven suspected human-to-human transmission events resulting in 42 apparent cases. However, they were unable to determine definitively if multiple introductions, either human or zoonotic, had occurred. They concluded the average household attack rate was 50% in an area undergoing a 600-fold increase of MPX cases, the highest ever reported [16]. There are no recorded cases of sustained human-to-human transmission with WA MPXV [6]. 

Identified risk factors for human-to-human transmission of MPX include sleeping in the same room/bed as an infected person, and activities that introduce the virus directly to the oral mucosa such as sharing the same plate and cups as an infected person [33]. 

Risk factors for zoonotic transmission of MPXV include living in forested or recently deforested areas [50]; no smallpox vaccination [50]; handling or eating dead bush meat or monkeys [49,51], and sleeping on the floor (in endemic areas) [33]. 

### 3.5. Prevention and Treatment

In 1979 the Global Commission for the Certification of Smallpox Eradication determined that smallpox vaccination to prevent MPX was not justified [5]. In 2010 a study comparing active surveillance data from a health zone in DRC from the 1980s and data from the same health zone in 2006/7 showed a 20-fold increase in MPX incidence. Furthermore, over 90% of the identified cases were born after the cessation of the smallpox eradication program, highlighting the impact of declining smallpox vaccination coverage [50]. In the 2003 USA epidemic the CDC recommended smallpox vaccination (ACAM2000™) up to 14 days post-MPXV exposure, for symptom reduction but not prevention of the disease [6,52]. The smallpox vaccine is currently not available to the public [6], nor used in MPXV endemic areas [41] due to concerns of cost, safety of using a vaccine containing live vaccinia virus, and the unknown effects of the vaccine in immunocompromised persons [16]. 

MPXV endemic countries are only found in sub-Saharan Africa, and this region of the world also accounts for 71% of the global burden of human immunodeficiency virus (HIV) [53]. Immunocompromised persons are at higher risk of serious vaccine complications, including progressive vaccinia (a rare but potentially fatal adverse event following smallpox vaccination causing progressive destruction of skin and tissue) [54,55] and potentially life-threatening side effects such as pneumonia and cryptococcal meningitis [56]. One of the prominent vaccines used in the smallpox global eradication campaign was Dryvax^®^ [57]. However, it caused concerning amounts of cardiac complications amongst recipients, and when used in immunocompromised persons major reactions were observed [58,59]. Due to these concerns, the end of the smallpox eradication campaign saw new replicating smallpox vaccine development. Those recommended by the CDC include the second generation vaccine ACAM2000™ (live attenuated vaccine originating from Dryvax^®^), and the further attenuated third generation modified vaccinia Ankara (MVA) vaccine, Imvamune [58]. Since 2007 the USA has been stock piling ACAM2000™ [57] and currently recommended it for post-exposure use in MPX cases (up to 14 days), to reduce symptoms but not necessarily prevent disease [6]. ACAM2000™ is similar to Dryvax^®^ in terms of immunogenicity, but unfortunately it also causes a similar frequency of cardiac adverse events [57]. Furthermore, its safety has not been tested in persons with HIV infection [56]. At the time of writing this paper, there is no specific advice from the CDC regarding smallpox vaccine in immunocompromised people who have been exposed to MPXV. However, since 2015 the CDC recommends that persons with HIV infection and CD4 cell counts of 50–199 cells/mm^3^ (those with CD4 cell counts <50 cells/mm^3^ might not benefit from smallpox vaccine [56]), who have been exposed to smallpox should be vaccinated with Imvamune (when antivirals are not available). If the CD4 count is above 200 cells/mm^3^ ACAM2000™ is recommended, as it is currently believed to be more effective (in animal studies of MPXV-infected monkeys, ACAM2000™ achieved complete viral suppression whereas Imvamune did not [58].) [56].

The need for a safer smallpox vaccine is evident, as currently if a mass vaccination were to take place, 1 in 145 persons vaccinated could develop cardiac complications such as myopericarditis [57] and, as cited by the WHO, in a country such as Germany with a population of 82 million, between 46 and 268 deaths could be expected, using the current second generation vaccines [58]. For this reason the CDC, WHO and Advisory Committee on Immunisation Practices (ACIP) do not recommend pre-event smallpox vaccination outside of certain identified groups including field investigators, veterinarians, animal-control and military personnel, and laboratory and health-care workers who are investigating, or are first-line responders at risk of, OPXV virus exposure [5,58,60]. 

Due to weaknesses of the currently available smallpox vaccines, exploration of other therapies such as immunoglobulin and antiviral therapies are of major importance in preventing severe or fatal OPXV infection amongst immunocompromised persons [54]. As of November 2016, two leading antiviral drugs were in development, namely ST-246 (Tecovirimat) and CMX001 (Brincidofovir, derived from the licensed antiviral drug cidofovir) [61]. Although stockpiled in the USA, the use of ST-246 as prophylaxis is still under Investigational New Drug (IND) status by the Food and Drug Administration (FDA) [6,62]. Categorised as a biodefence product, the treatment for therapeutic use is in phase III trials and development for use as an adjunct with smallpox vaccines (to prevent disease and reduce vaccine-related complications) is at pre-clinical trial stage [62]. The use of antivirals for treatment of OPXV disease in animal studies have proven successful, with no major adverse events. In 2010 a randomized double-blind, placebo-controlled study was conducted with inoculating a lethal dose of MPXV into cynomolgous macaques, and found the use of ST-246 three or four days post-infection not only protected animals from deadly infection, but also reduced lesion formation and viral DNA levels in the blood [54,63]. So far, Phase I clinical trials have shown that ST-246 is a safe therapeutic for treating OPXV infections in humans (in the early stages), and even prevents disease if given in the incubation period [54,63]. During the USA outbreak the CDC recommended the use of ST-246, but are still further establishing the efficacy of its use treating MPXV in humans [6]. Development of guidance documents for the use of antivirals for OPXV by the CDC was in progress at time of writing [56].

With regards to immune globulin, the CDC only recommends vaccinia immune globulin (VIG) for prophylactic use in severely immunodeficient persons exposed to MPXV, as there has been no proven benefit in its treatment of smallpox complications [6]. Like ST-246, VIG also remains under IND category [6]. Advances in vaccinations and antiviral treatments since the eradication of smallpox is encouraging. 

Further development of third generation MVA vaccines including ACAM3000 and TBC-MVA is ongoing [58], and clinical trials continue for antiviral therapies. Ultimately, until these advances become available to those living in remote endemic environments, prevention of MPX requires reduced contact with infected animals and prevention of human-to-human transmission through isolation and basic hygiene [33,41]. Film-based educational activities have been effective in MPX awareness in DRC [39], but further health education campaigns focused on the handling of possible animal reservoirs is needed [50].

## 4. Discussion

The smallpox-like rash and classic prodromal symptoms can make MPX difficult to differentiate from other pox-like illness. However, marked lymphadenopathy and lesions on mucosa, palms and soles, are key distinguishing clinical features of MPX. The USA epidemic deepened our clinical understanding of MPX, revealing biochemistry findings that were previously difficult to obtain, and stimulating new research. Yet we currently have an incomplete understanding of MPXV transmission [1]. 

It is unknown if the animal species endemic to the USA can maintain a zoonotic cycle of MPXV [5]. However, it is believed repeated animal reintroduction of MPXV is required to sustain the disease in the human population [64]. Despite the importance of reservoir species in transmission, studies suggest the survival of MPXV is affected by environmental conditions [9]. Common theories include: (i) specific temperature and light conditions increase the time MPXV survives outside a host [9]; (ii) deforestation and flooding could increase habitats for species carrying MPXV, causing increased frequency and contact thus risk of transmission [9], as seen in the 2005 Sudan flooding and subsequent MPX outbreak [21]; and (iii) expansion of the rainforest driven by warmer and more humid conditions may allow MPXV and its reservoirs to also expand their geographic range, potentially leading to accelerated dissemination of the virus [65]. 

As human-to-human transmission appears to be limited, the majority of MPX cases are closely linked with spill-over transmission from animal reservoirs, thus the geographic range of MPX will be influenced by the habitat of the reservoir species [45,50]. If establishment of MPXV in a reservoir outside of Africa is possible, the global public health setback would be considerable [19]. This would result in a lost opportunity to combat the infection while its current geographic territory is limited [50].

Surveillance of MPX is difficult due to limited resources and infrastructure, inappropriate diagnostic material, difficulties in accessing conflict areas [9,41], and lack of clinical recognition of MPX [13,45]. Awareness of the key clinical characteristic of MPX will aid clinical detection. Moreover, development of easy-to-use rapid tests in combination with simple diagnostic algorithms [3] would aid diagnosis and thereby the containment of an MPX epidemic [5].

## 5. Conclusions

Smallpox vaccination has allowed for relatively low MPX incidence [9]. However, a younger, non-immune generation combined with populations dependent on hunting for food have resulted in re-emergence [28,50]. Smallpox vaccination is contraindicated in immunodeficient individuals. Further development of MVA vaccines is underway, but full licensure of antiviral treatment options to compliment prevention activities should be considered [61]. Avoidance to do so implies ignoring MPX morbidity and mortality currently endured by indigenous populations most notably in endemic DRC [50].

The USA epidemic proved MPXV has the capacity to infect and cause high levels of morbidity in a range of hosts worldwide [11,50]. Had it been the more aggressive CB clade a high mortality may have ensued [14]. Globalisation means countries must not ignore what were once considered geographically-restricted infectious agents [5]. Improved surveillance is needed for strategies to target transmission reduction [50] and increase knowledge of disease burden [13]. The sylvatic component of the cycle means eradication is not possible [28], therefore prevention becomes paramount. In light of environmental impacts, further research to identify the reservoir host or hosts, and targeted educational programmes, are necessary to protect those most vulnerable.

## Figures and Tables

**Table 1:** Core Clinical Features of Human Monkeypox.

Specific Symptoms	Primary Research ^1^	Secondary Research ^2^
Fever and Fatigue	Huhn et al. (2005) [35] Reed et al. (2004) [19] Reynolds et al. (2006) [29] Formenty et al. (2010) [21] Jezek et al. (1987) [32]	Bavari & Whitehouse (2005) [28] Macneil et al. (2009) [3] Sale et al. (2006) [15] Di Giulio & Eckberg (2004) [5] Nolen et al. (2015) [33] Parker et al. (2007) [14]
Rash	Huhn et al. (2005) [35] Reed et al. (2004) [19] Reynolds et al. (2006) [29] Formenty et al. (2010) [21] Jezek et al. (1987) [32]	Bavari & Whitehouse (2005) [28] Macneil et al. (2009) [3] Sale et al. (2006) [15] Di Giulio & Eckberg (2004) [5] Nolen et al. (2015) [33] Parker et al. (2007) [14] Breman (2000) [34]
Lymphadenopathy	Huhn et al. (2005) [35] Reed et al. (2004) [19] Reynolds et al. (2006) [29] Formenty et al. (2010) [21] Jezek et al. (1987) [32]	Bavari & Whitehouse (2005) [28] Macneil et al. (2009) [3] Sale et al. (2006) [15] Di Giulio & Eckberg (2004) [5] Nolen et al. (2015) [33] Parker et al. (2007) [14]
Lesions (including palms of hands and soles of feet)	Huhn et al. (2005) [35] Reed et al. (2004) [19] Reynolds et al. (2006) [29] Formenty et al. (2010) [21] Jezek et al. (1987) [32]	Bavari & Whitehouse (2005) [28] Macneil et al. (2009) [3] Sale et al. (2006) [15] Di Giulio & Eckberg (2004) [5] Nolen et al. (2015) [33] Parker et al. (2007) [14]
Respiratory symptoms	Reed et al. (2004) [19] Reynolds et al. (2006) [29] Formenty et al. (2010) [21] Jezek et al. (1987) [32]	Parker et al. (2007) [14] Di Giulio & Eckberg (2004)

^1^ Denotes original studies and research papers. ^2^ Denotes collation/synthesis of existing research.

**Table 2:** Recorded Case Fatality Rate of Human Monkeypox 1970–2005.

Date and Location	1970–1979 Central and West Africa	1981–1986 DRC	1996–1998 DRC	2003 USA	2005 South Sudan
Case Fatality Rate (%)	17 [40]	9.8 ^1^ [41,42,43]	1.5 ^2^ [44]	No recorded deaths	No recorded deaths

^1^ Specifically between 1981 and 1985 the recorded CFR was 9% [43]; ^2^ The low CFR between 1996 and 1997 was suggestive of varicella not MPXV [28].

**Table 3:** Suspected Reservoir Host of Monkeypox Virus.

Suspected Reservoir Host	Primary Research	Secondary Research
Rope squirrel (*Funisciurus* sp.)	Fuller et al. (2011) [45] Thomassen et al. (2013) [9] Khodakevich et al. (1986) [46]	Guarner et al. (2004) [18] Sale et al. (2006) [15] Di Giulio & Eckberg (2004) [5] Parker & Buller (2013) [11]
Gambian pouched rat (*Cricetomys gambianus*)	Hutson et al. (2015) [1]	Parker & Buller (2013) [11] Sale et al. (2006) [15] Fuller et al. (2011) [45] Di Giulio & Eckberg (2004) [5] Formenty et al. (2010) [21]
Sooty mangabey monkey (*Cercocebus atys*)	Radonic et al. (2014) [4]	Nolen et al. (2015) [33]

**Table 4:** Diagnostic Tests for Monkeypox or Orthopoxvirus.

Test	Description
Viral culture/isolation	Live virus is grown and characterised from a patient specimen
Electron microscopy	Clear image of a brick-shaped particle for visual classification of a poxvirus
Immunohistochemistry	Tests for the presence of OPXV specific antigens
PCR (including real-time PCR)	Tests for the presence of MPXV specific DNA signatures
Anti-OPXV IgG	Tests for the presence of OPXV antibodies
Anti-OPXV IgM	Tests for the presence of OPXV antibodies
Tetracore OrthopoxBioThreat	Alert test for the presence of OPXV antigens

Source: adapted from McCollum & Damon (2014) [13]: “Diagnostic tests for monkeypox or orthopoxvirus”.

**Table 5:** Modes of Transmission of Monkeypox Virus.

Transmission	Primary Research	Secondary Research
Direct contact with infected humans or animals	Guarner et al. (2004) [18] Jezek et al. (1988) [7] Meyer et al. (2002) [49] Reed et al. (2004) [19] Reynolds et al. (2006) [29] Learned et al. (2005) [22] Formenty et al. (2010) [21]	Rimoin et al. (2010) [50] Parker & Buller (2013) [11] Sale et al. (2006) [15] Hammarlund et al. (2005) [8] Hutson et al. (2015) [1] McCollum & Damon (2014) [13]
Respiratory	Guarner et al. (2004) [18] Hammarlund et al. (2005) [8] Reynolds et al. (2006) [29]	Parker & Buller (2013) [11] Hutson et al. (2015) [1]
Fomites	Hammarlund et al. (2005) [8] Nolen et al. (2015) [33] Reynolds et al. (2006) [29] Formenty et al. (2010) [21]	Parker & Buller (2013) [11]
Consuming infected meats	Meyer et al. (2002) [49] Nakouné E, Kazanji M (2012) [51]	Parker & Buller (2013) [11] Sale et al. (2006) [15] Thomassen et al. (2013) [9]
